# Prostate-Specific Membrane Antigen (PSMA): A Potential Theranostic Biomarker in Breast Cancer

**DOI:** 10.3390/biomedicines14030628

**Published:** 2026-03-11

**Authors:** Alessandra Virga, Flavia Foca, Stefania Cortecchia, Francesca Poli, Paola Caroli, Federica Matteucci, Roberta Maltoni, Massimiliano Mazza, Fabio Nicolini, Paola Ulivi, Giovanni Paganelli, Maurizio Puccetti, Sara Bravaccini

**Affiliations:** 1 Biosciences Laboratory, IRCCS Istituto Romagnolo per lo Studio dei Tumori (IRST) “Dino Amadori”, 47014 Meldola, Italy; alessandra.virga@irst.emr.it (A.V.); massimiliano.mazza@irst.emr.it (M.M.); fabio.nicolini@irst.emr.it (F.N.); paola.ulivi@irst.emr.it (P.U.); 2 Unit of Biostatistics and Clinical Trials, IRCCS Istituto Romagnolo per lo Studio dei Tumori (IRST) “Dino Amadori”, 47014 Meldola, Italy; 3 Azienda Unità Sanitaria Locale (AUSL) Imola, 40026 Imola, Italy; s.cortecchia@ausl.imola.bo.it (S.C.); f.poli@ausl.imola.bo.it (F.P.); puc56@libero.it (M.P.); 4 Nuclear Medicine Unit, IRCCS Istituto Romagnolo per lo Studio e la Cura dei Tumori (IRST) “Dino Amadori”, 47014 Meldola, Italy; paola.caroli@irst.emr.it (P.C.); federica.matteucci@irst.emr.it (F.M.); giovannipaga55@gmail.com (G.P.); 5 Department of Medical Oncology, IRCCS Istituto Romagnolo per lo Studio dei Tumori (IRST) “Dino Amadori”, 47014 Meldola, Italy; roberta.maltoni@irst.emr.it; 6 Department of Medicine and Surgery, University of Enna “Kore”, 94100 Enna, Italy; sara.bravaccini@unikore.it

**Keywords:** breast cancer, PSMA expression, theranostic biomarker, TILs, Ki67 and PSMA PET/CT

## Abstract

**Background**: Subtype classification for breast cancer (BC) patients is important for risk-stratification. Unfortunately, this parameter is not always able to discriminate between high- and low-risk diseases. Glutamate Carboxypeptidase-II (GCPII), also known as prostate-specific membrane antigen (PSMA), could be an important biomarker of aggressiveness, given that it has been reported to be expressed in BC tumor cells and even more in endothelial cells of tumor vessels. **Methods**: We analyzed 22 Luminal A, 47 Luminal B, 9 HER2-positive (HER2+), and 23 triple-negative (TN) BC to assess whether PSMA, Ki67 expression, and tumor-infiltrating lymphocytes (TILs) were different in BC subtypes. **Results**: Median PSMA and Ki67 values were significantly higher in TNBC than in Luminal A and B tumors. We saw a correlation between PSMA and Ki67 expression, especially in HER2+ tumors (*p* = 0.035), while an inverse correlation between PSMA and TILs was observed in Luminal A (*p* = 0.028). **Conclusions**: Our results suggest that PSMA could be used as a biomarker in BC, given that it is highly expressed in more aggressive tumors. These findings open the way to a clinical investigation for the possible use of PSMA as a theranostic biomarker in BC patients with PSMA positive PET scan.

## 1. Introduction

Breast cancer (BC) is the leading cause of cancer-related death among women worldwide. Recent global cancer statistics indicate that BC has surpassed lung cancer as the most commonly diagnosed cancer, with an estimated 2.3 million new cases in 2020. Besides being the most common cancer, BC, responsible for approximately 685,000 deaths globally [1], is a heterogeneous disease with a multifaceted etiology that includes a wide range of tumors with different morphological, biological, and clinical characteristics [2]. BC treatments depend upon the patient and tumor characteristics, such as the clinical stage and grade of the disease, the histopathological features, and the status of biomarkers (estrogen receptor (ER) and progesterone receptor (PR), human epidermal growth factor receptor 2 (HER2), and Ki67 expression). Imaging of the breast is primarily used for the detection, diagnosis, and clinical management of cancers [3]. ESMO guidelines recommend performing bilateral mammograms and ultrasound on both breasts and lymph nodes, along with a core biopsy [4]. Only in specific cases (patients diagnosed with early-stage, ER-positive, HER2-negative invasive BC) is gene expression profiling necessary to predict whether BC will recur within 10 years from diagnosis [5]. The most frequently used gene expression tests in patients with hormone receptor-positive, HER2-negative, node-negative or limited-node-positive BC are Oncotype DX and PAM50 [6], which permit BC classification into different intrinsic molecular subtypes based on 21 or 50-gene expression profiles, respectively [7,8]. Each subtype exhibits distinct clinical and biological characteristics that influence patient outcomes [9]. In current clinical practice, immunohistochemistry (IHC) is the only assay performed to assess the conventional biological biomarker status (hormone receptors, HER2, Ki67), and the subtype classification obtained with this technique is considered a surrogate classification of the molecular subtype of tumors [10].

These subtypes are commonly grouped by pathologists into four categories—Luminal A, Luminal B, HER2-positive (HER2+), and triple-negative breast cancer (TNBC)—with the latter being characterized by the lack of expression of ER, PR, and HER2 receptors [11].

Despite the importance of subtype classification obtained by IHC for BC patients in terms of risk stratification and treatment choice, this evaluation is not able to discriminate perfectly between high- and low-risk disease, and the identification of an accurate biomarker of tumor evolution is still a clinical need [12,13,14,15,16,17,18,19].

Among emerging biomarkers for BC, prostate-specific membrane antigen (PSMA) has gained attention as a potential therapeutic target due to its overexpression on the vasculature of many solid tumors, and not only in prostate cancer, where its value has already been established [20]. PSMA is an important biomarker because it has been reported to be expressed in the neoformed vessels of the tumors [21,22] and in some cases also in BC epithelial cells [20,23,24]. PSMA expression has been reported both in primary tumor cells and in distant metastases, suggesting that it may be a suitable target for antiangiogenic therapies across different tumor types. PSMA has been shown to be a target for PET imaging for prostate cancer, at primary staging and biochemical relapse [21,22].

Heesch et al. highlighted the potential for [177Lu] Lu-PSMA radioligand therapy as an emerging therapeutic approach for BC treatment [25]. It is well known that stromal tumor-infiltrating lymphocytes (TILs) are well-established, prognostically favorable clinicopathologic biomarkers in HER2+ and TNBC tumors [26,27,28,29], and that Ki67 reflects BC tumor aggressiveness [30,31]. In our study, they were used as standard biological markers to contextualize tumor aggressiveness within the cohort and as comparative reference markers for evaluating PSMA expression.

For these reasons, we decided to analyze the role of PSMA expression in different BC subtypes with different tumor aggressiveness in relation to Ki67 expression and TIL presence to determine whether PSMA expression could represent a suitable target for antiangiogenic therapy and radionuclide treatment.

## 2. Materials and Methods

### 2.1. Methods Case Series

This retrospective study included 101 patients with BC followed at the Local Health Unit of Imola, Italy, from 2015 to 2023. Among these, 22 (21.8%) were Luminal A, 47 (46.5%) were Luminal B, 9 (8.9%) were HER2+, and 23 (22.8%) were TNBC. Overall, 6 biopsies and 95 primary tumors were analyzed. The study protocol was reviewed and approved by the Institutional Review Board of IRCCS Istituto Romagnolo per lo Studio dei Tumori (IRST) “Dino Amadori”, Meldola, Italy (study IRST100.34 approved on 17 May 2016). All the analyses were carried out in accordance with the relevant guidelines and regulations.

Despite all reasonable efforts to obtain informed consent for participation in the study, this was not feasible, as all patients had either passed away or were lost to follow-up. Formalin-fixed paraffin-embedded samples were used for diagnosis. The histology and grading of breast lesions were established by expert pathologists at the Local Health Unit of Imola. The study was conducted in compliance with the Declaration of Helsinki and the Good Clinical Practice Guidelines of the International Conference on Harmonization.

### 2.2. Immunohistochemistry

The Ventana BenchMark ULTRA staining system was used to assess the immunostaining for the conventional biomarkers and PSMA expression (Ventana Medical Systems, Tucson, AZ, USA) with Optiview DAB Detection Kit (Ventana Medical Systems). Slides with tissue sections were incubated with ready-to-use anti-PSMA antibody (EP192 Rabbit Monoclonal-Cell Marque Corporation, Rocklin, CA, USA) for 1 h. Sections were automatically counterstained with hematoxylin II (Ventana Medical Systems). As negative and positive controls, healthy breast tissues and prostate cancer tissues were used, respectively, in all the experiments.

The percentage of tumor cells with nuclear staining out of the total number of tumor cells was used to quantify ER (SP1 Rabbit Monoclonal-Ventana^®^), PR (1E2 Rabbit Monoclonal-Ventana^®^), and Ki67 (30-9 Rabbit Monoclonal-Ventana^®^) expression in 10 fields at 40x magnification, while for HER2 (4B5 Rabbit Monoclonal-Ventana^®^), only membrane positivity was taken into account. Stromal tumor-infiltrating lymphocytes (TILs) were quantitatively detected on a hematoxylin–eosin-stained section by microscope, evaluating the percentage of TILs according to the Salgado guidelines [32] in 10 fields at 40x magnification to obtain a comprehensive assessment of the sample. PSMA positivity was calculated as the number of positive vessels in 10 fields at 40x magnification. All samples were evaluated by 2 independent observers. Discrepancies in vessel count greater than 10% were considered a disagreement and were resolved by consensus after joint review using a multihead microscope.

### 2.3. Statistical Analysis

To describe the data, summary statistics such as the median and range or interquartile range (iqr) were reported. The Kruskal–Wallis test was used to assess the difference in PSMA distribution among BC subtypes, and Dunn’s test was performed for post hoc comparisons; Spearman’s rho coefficient was calculated to analyze the correlation among PSMA, Ki67, and TILs. The median value of biomarkers was considered for the analysis. All statistical analyses were performed using Stata/SE version 15.1 for Windows (StataCorpLP, College Station, TX, USA).

Due to the lack of survival data in our patient case series, we queried public online databases to explore the relationship between *FOLH1* (PSMA), *MKI67* (Ki67) gene expression and overall survival using RNA expression data. The Human Protein Atlas (HPA) database survival module was queried for *FOLH1* expression in breast invasive carcinoma [33]. Additionally, the KM plotter tool (v2.0) [34] was used with default parameters (median cutoff, RNA expression data, no probe-set filtering) to generate overall survival curves for *FOLH1* and *MKI67* in a cohort of patients with BC. Hazard ratios, log-rank *p*-values, and 95% CIs were considered.

## 3. Results

### 3.1. Results from Our Study Cohort

The median age at diagnosis of BC patients was 66 (min-max: 25–87 years). Seventy (69.3%) patients had ER > 0% and 64 (63.4%) had PR > 0%. The ductal histotype was present in 92 patients (91.0%). Most of the patients had grade 3 tumors (55.4%). The tumor size was from 1 to 1.99 cm for 37.5% of the cases (Table 1).

The median PSMA values, in terms of the average number of positive vessels for each patient, were higher in TNBC compared to Luminal A and B tumors (*p* < 0.05) (Table 2).

**Figure 1:**
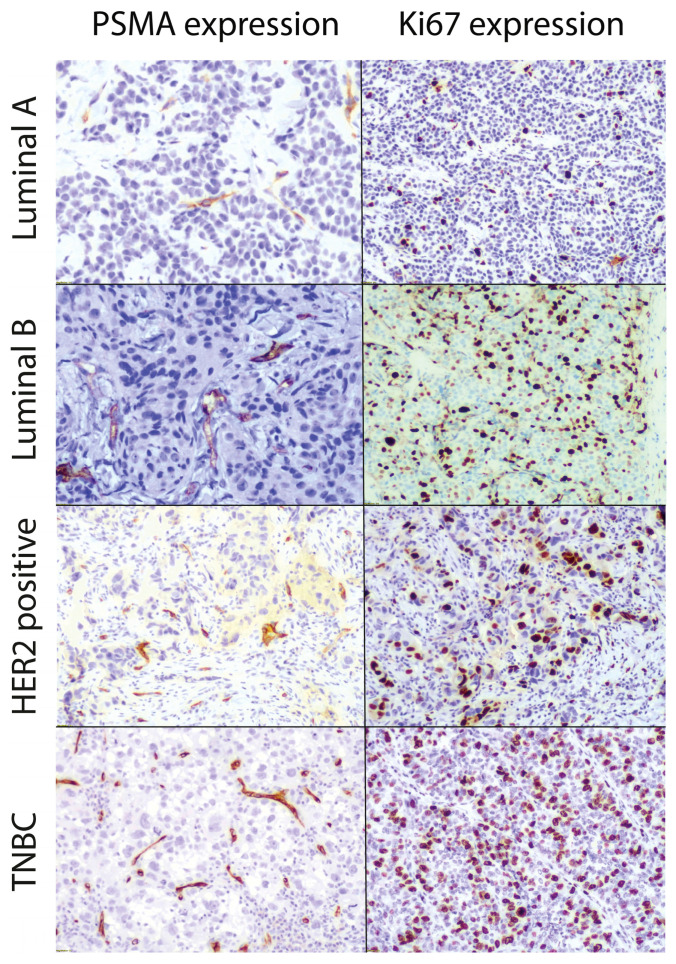
PSMA and Ki67 IHC analysis. PSMA and Ki67 expression in the different breast cancer subtypes (10x magnification).

Figure 1 shows the expression of PSMA and Ki67 in different tumor subtypes. The former was expressed only in the neovessels of the tumor, while the latter was expressed in the nucleus of tumor cells.

**Figure 2:**
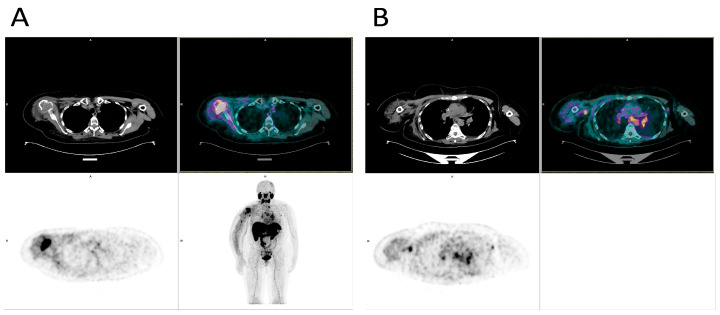
The figure shows 68Ga-PSMA PET/CT images illustrating in vivo PSMA expression in a 46-year-old female patient with metastatic triple-negative breast cancer (TNBC). (**A**) The upper section shows details of the lesion in the right humerus in CT and CT-PET fusion scan, while the lower section presents PET images and the Maximum Intensity Projection (MIP) image, with the image at the bottom right reflecting the whole body. (**B**) The upper section again presents the lesion in the left axilla in CT images and CT-PET fusion scan, while the lower section shows the PET image and MIP image, with the image at the bottom right reflecting the whole body.

Figure 2 shows 68Ga-PSMA PET/CT images of in vivo PSMA expression in a 46-year-old female patient with metastatic TNBC (Ki67 80%) who underwent neoadjuvant chemotherapy and bilateral mastectomy in 2018. These images of this single case are representative of multiple patients involved in this study. A Ga-PSMA PET scan performed in September 2023 was conducted to evaluate receptor expression prior to potential [177Lu] Lu-PSMA therapy. The scan reveals several areas of pathological hyperaccumulation of the radionuclide, with significant findings in the left axillary adenopathy and right humerus.

**Figure 3:**
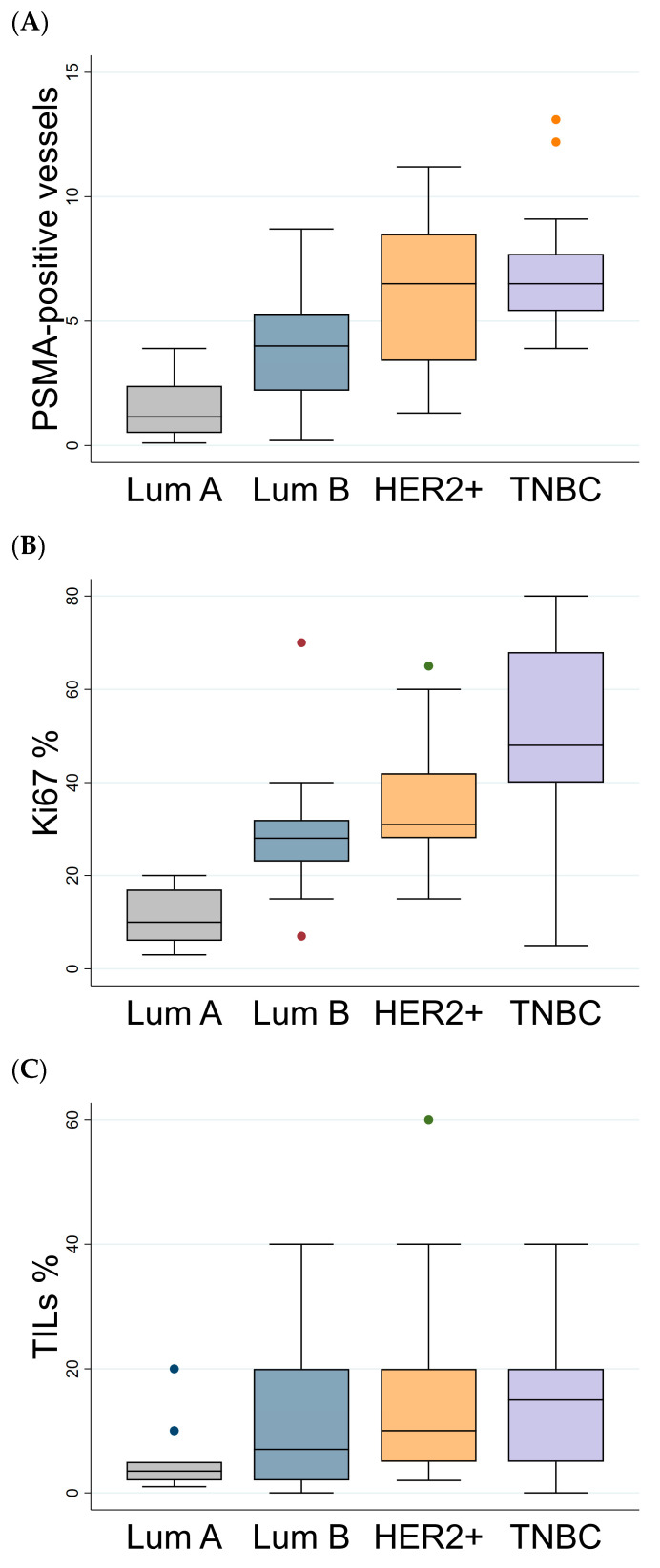
Box plots of PSMA, Ki67, and TILs in the different BC subtypes. Distribution of PSMA (**A**), Ki67 (**B**) expression, and TILs (**C**) in the different subtypes (Luminal A, Luminal B, HER2+, and TNBC). Lum: Luminal; TNBC: triple-negative breast cancer; TILs: tumor-infiltrating lymphocytes.

The distribution of PSMA, Ki67 expression, and TIL values was significantly different in the analyzed subtypes (Table 2). Notably, HER2+ tumors exhibited significantly higher PSMA values (median 6.5; iqr 3.4–8.5) compared to Luminal B tumors (median 4.0; iqr 2.2–5.3; *p* = 0.026). Differences were observed in PSMA expression between Luminal B vs. Luminal A (*p* < 0.001), HER2+ vs. Luminal A (*p* < 0.001), HER2+ vs. Luminal B (*p* = 0.026), and TNBC vs. Luminal A (*p* < 0.001), TNBC vs. Luminal B (*p* < 0.001) (Table 2 and Figure 3A). Ki67 was higher in TNBC compared to Luminal A, Luminal B, and HER2-positive tumors, even if the latter comparison was not statistically significant (*p* < 0.001, *p* < 0.001, and *p* = 0.068) (Table 2 and Figure 3B). Finally, the number of TILs was significantly lower in Luminal A compared to Luminal B, HER2+, and TNBC (*p* = 0.018, *p* = 0.009, and *p* = 0.005, respectively) (Table 2 and Figure 3C).

**Figure 4:**
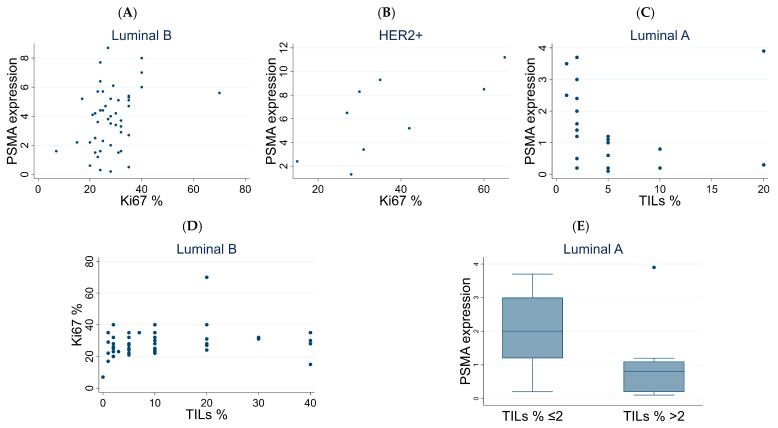
Correlation analysis between PSMA, Ki67, and TILs. (**A**) Correlation between PSMA and Ki67 expression in Luminal B subtype. (**B**) Correlation between PSMA and Ki67 expression in HER2+ subtype. (**C**) Correlation between PSMA expression and TILs in Luminal A subtype. (**D**) Correlation between Ki67 expression and TILs in Luminal B subtype. (**E**) PSMA expression in patients with TILs <= 2 vs. TILs > 2 (only in Luminal A subtype). TILs: tumor-infiltrating lymphocytes.

PSMA and Ki67 were characterized by a positive correlation, in particular in the HER2+ (*p* < 0.035) and in the Luminal B (*p* = 0.039) subtypes, while a negative correlation between PSMA and TIL expression was observed in Luminal A (*p* = 0.028) (Table 3 and Figure 4A–C). A positive correlation between Ki67 and TILs was also observed in the Luminal B subtype (Table 3 and Figure 4D). In particular, we tested the correlation between PSMA expression and TILs categorized as TILs <= 2 vs. TILs > 2 in the Luminal A subtype. PSMA expression was higher in TILs <= 2 than in TILs > 2 (*p* = 0.002) (Figure 4E).

**Figure 5:**
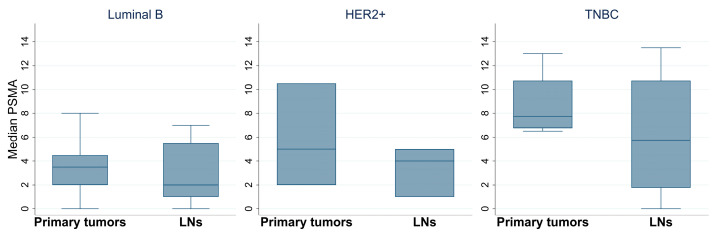
Box plots of PSMA expression in primary tumors and lymph nodes. PSMA expression on primary tumors and lymph nodes in Luminal B, HER2+, and TNBC subtypes. TNBC: triple-negative breast cancer, LNs: lymph nodes.

The analysis of PSMA expression on the primary tumors and lymph nodes was available only for a subgroup of patients in Luminal B, HER2+, and TNBC tumors (n = 7, 3, and 4 patients, respectively). PSMA median values were similar between the primary tumor and metastatic site for all subtypes (Figure 5).

### 3.2. Public Database Data

We explored the relationship between *FOLH1* (PSMA), *MKI67* (Ki67) gene expression, and survival in the HPA public database. No significant prognostic correlation between *FOLH1* expression and overall survival was observed in invasive BC patients (p-value from log-rank test: 0.16; Supplementary Figure S1), while an inverse relationship between *MKI67* and overall survival was shown (*p*-value = 0.0011, Supplementary Figure S2).

## 4. Discussion

The heterogeneity of BC, which is characterized by diverse morphological, biological, and clinical features, requires a targeted and personalized approach for diagnosis and treatment. Current clinical practice is based on patient and tumor characteristics such as clinical stage, histopathological features, and biomarker status to guide patients’ follow- up and treatment [2,3]. Despite the classification of BC subtypes being crucial for patient management and risk assessment, the differentiation between high- and low-risk disease in terms of relapse is not completely accurate. Therefore, the search for an ideal theranostic biomarker is still an unmet clinical need. Among the emerging biomarkers for BC, PSMA has attracted interest as a potential therapeutic target due to its overexpression in the neovasculature of various solid tumors [20]. Moreover, given the possibility of treating patients with radiolabeled compounds, the assessment of PSMA expression is even more appealing. In the present study, we aimed to evaluate the potential role of PSMA in all BC subtypes, with different prognoses: Luminal A, Luminal B, TNBC, and HER2+ tumors. In particular, we considered HER2+-only patients with expression of HER2 without hormone receptor expression, while those with hormone receptor expression were included in the Luminal B subtype for their different prognosis.

In comparison with data reported in the literature [35], we observed a higher presence of tumors with ductal histotype and of grade 3, reflecting the high aggressiveness of the disease in our population of patients. This strongly indicates that the study population represents a subgroup of patients characterized by unfavorable outcomes, rather than the general BC population. Some authors reported the presence of PSMA positivity in healthy and benign tissues [36,37,38]. In accordance with our previous observations [21], the expression of PSMA was present in the neovessels of the tumor only, and its levels were significantly higher in TNBC compared to Luminal A and Luminal B tumors. The expression of PSMA has been identified not only in primary BC but also in distant metastases of the same patient when available [24]. In line with our findings, Wernicke AG and colleagues saw PSMA expression both in the primary tumors and in the metastatic sites [24].

Researchers have shown that more aggressive tumors, characterized by lower differentiation and higher grades, have high PSMA expression [20,39].

Overall, in this study, the subtype-specific distribution of PSMA expression reflects the distinct biological behavior of breast cancer subtypes, mirroring the more aggressive phenotype of TNBC compared with Luminal tumors. The relatively lower PSMA expression observed in Luminal A tumors may have relevant clinical implications. Given that Luminal A tumor represents the least aggressive BC subtype, we expected lower PSMA expression in this group, considering its association with neoangiogenic activity and tumor aggressiveness [38]. However, PSMA overexpression within Luminal A tumors could identify a subset of patients with a more aggressive biological behavior and a potentially higher risk of recurrence, including late recurrence, likely reflecting increased angiogenic activity [40].

In the subtype of the Luminal B tumors, the expression of PSMA is even more important, considering the clinical necessity to distinguish the more aggressive lesions in clinical practice [41]. Up to now, different genomic profiling tests are used for BC patients with intermediate Ki67 to establish the recurrence risk of relapses within 10 years from the diagnosis of the primary tumor. Unfortunately, these tests are used only in patients with hormone receptor-positive, HER2-negative, node-negative, or limited-node-positive BC, and the potential theranostic role of PSMA [6] in this subtype of patients is even more attractive. The luminal subtypes of BC, which are characterized by the presence of steroid hormone receptors, typically have a more favorable prognosis and reduced proliferation rates compared to the other BC subtypes [42]. The correlation between PSMA expression and tumor proliferation is further emphasized by the observation that higher Ki67 levels were also found in TNBC compared to other subtypes, reinforcing the association between elevated PSMA expression and increased tumor aggressiveness [43]. Considering the different prognosis of HER2+ patients according to the presence or absence of hormone receptors, we considered as HER2+ patients only those without hormone receptor expression, given their well- known unfavorable prognosis [44]. The higher expression of PSMA in HER2+ tumors than in Luminal B tumors reflects the different biological behavior of HER2+ tumors when compared to hormone receptor-positive Luminal B cancers. Although Luminal B tumors are characterized by higher proliferation rates than Luminal A tumors, HER2+ tumors lacking hormone receptor expression demonstrated PSMA levels more comparable to TNBC. In particular, while Luminal B tumors retain hormone receptor expression and typically exhibit intermediate proliferation rates, HER2+ tumors without hormone receptor expression are characterized by enhanced HER2-driven signaling pathways, increased angiogenic activity, and more aggressive clinical behavior. The higher PSMA expression observed in HER2+ tumors may therefore be biologically linked to increased neovascularization and tumor metabolic demand, reinforcing that PSMA expression may reflect tumor aggressiveness more closely than conventional subtype classification alone.

The strong correlation between PSMA and Ki67 in HER2+ tumors may offer a new perspective, opening up the possibility of using novel combined approaches, with anti-HER2 drugs and radiolabeled compounds [45,46].

Despite the study limitations, such as the limited number of cases analyzed, the lack of a universal PSMA cut off value to distinguish between PSMA positive and negative tumors, and the lack of complete follow-up data, we successfully investigated the expression of PSMA across various BC subtypes, with particular attention to TNBC, given the lack of targeted therapies for the patients with this tumor subtype. We explored the expression of PSMA in the different tumor subtypes in relation to Ki67 expression and stromal TILs, given their well-established clinical prognostic role. As expected, we observed the highest expression of PSMA and Ki67 in TNBC and HER2+ subtypes, and this reflects the high aggressiveness of the disease, considering that in both subtypes, patients are hormone receptor-negative and have poor prognosis.

Future validation in larger, independent cohorts using standardized PSMA IHC scoring will be essential to clarify the biological heterogeneity of PSMA expression across BC subtypes. Moreover, prospective PSMA PET/CT studies with paired tissue sampling will be crucial to elucidate the mechanistic relationship between imaging, PSMA expression, and microenvironmental features. As datasets with adequate longitudinal follow-up become available, outcome-driven cutoff refinement (including ROC-based approaches) will help define clinically meaningful thresholds of PSMA expression.

Public databases analyses on *FOLH1* (PSMA) and *MKI67* (Ki67) gene expression and invasive BC patients’ overall survival revealed no significant association for *FOLH1* expression. In contrast, high *MKI67* expression was inversely related to poor survival. These external validations partially corroborate our IHC results while underscoring challenges in correlating mRNA and protein data with survival. Further studies of PSMA IHC in larger cohorts of BC patients with follow-up data are warranted.

In parallel, early-phase clinical studies investigating PSMA-targeted radioligand therapy in PSMA-expressing BC, particularly in triple-negative tumors, should incorporate PSMA PET both for patient enrichment and for assessing target engagement and therapeutic response, thereby strengthening the translational bridge between imaging-defined phenotypes and biological behavior.

As expected, TIL levels were significantly lower in Luminal A than in other breast cancer subtypes, particularly the highly inflamed TNBC [32].

Subsequent research efforts on the interplay between TILs and PSMA, taking into consideration also the localization of TILs within the tumor in relation to PSMA expression, could be useful to understand the role of immune evasion mechanisms in BC. For several tumors, the use of PSMA-directed therapy especially when combined with immunotherapy, has shown preliminary preclinical and clinical evidence of potential benefit [47,48].

PSMA and TIL expression showed a significant correlation in the overall series, but, unexpectedly, with an inverse association in the Luminal A subtype. For deeper analysis, we investigated PSMA expression associated with categorized TILs < 2 and TILs >= 2 in Luminal A tumors. Our findings showed significantly higher PSMA expression in TILs < 2 and lower in TILs >= 2. TILs have been shown to provide a prognostic and potentially predictive role, as reported in the recommendations formulated by the International Working Group on TILs in 2014 and published in 2015 [32,49]. Few data in the literature are present on the relation between PSMA expression and TILs. It is well known that Luminal A patients can relapse after 10 years from the first diagnosis [50]. We believe that Luminal A patients with higher levels of PSMA expression should be monitored over a long follow-up period to determine whether PSMA has a prognostic role in this subtype.

The identification of PSMA as a potential biomarker for aggressive BC subtypes can open up the possibility of new treatment options in clinical practice, considering the possibility of using PSMA PET/CT imaging for the evaluation of eligibility for anti-angiogenic agents and PSMA radionuclide therapy with alpha or beta emitter isotopes. Researchers have demonstrated that PSMA uptake was higher in TNBC, which may be the best candidate for PSMA-based PET-CT radionuclide imaging. Given the limited therapeutic options available for these patients, PSMA-targeted therapy may represent an innovative treatment approach that merits further investigation [20]. In addition, our research group previously demonstrated a correlation between PSMA expression and PSMA uptake by PSMA PET/CT [21,22] in other tumor types. These data highlight the importance of PSMA expression also in BC patients, and this biomarker may represent a new diagnostic and therapeutic option in patients with PSMA expression in the neovessels of the tumor. The findings of this study highlight the importance of PSMA as a potential biomarker across various BC subtypes, particularly in identifying the more aggressive forms of the disease, such as TNBC and HER2-positive tumors. This is particularly relevant given the current limitations for targeted therapies for TNBC patients and the need for effective biomarkers that can guide treatment decisions. Moreover, the correlation between PSMA expression and other markers of aggressiveness, such as Ki67 and TILs, highlights the multifaceted nature of BC biology [6,7].

## 5. Conclusions

PSMA could represent a potential theranostic biomarker, given the availability of PSMA PET/CT techniques. Given the role of PSMA as a biomarker of aggressiveness, patients with high PSMA expression, including those with Luminal A tumors, should undergo prolonged follow-up to clarify its clinical significance. Future research should focus on elucidating the mechanisms underlying PSMA expression and its interactions with other biomarkers to further refine patient stratification and therapeutic interventions.

## Figures and Tables

**Table 1:** Patient characteristics (n = 101).

Patient Characteristics	Tumor Subtype				Tot Patients N (%)
	Luminal A (n = 22)	Luminal B (n = 47)	HER2+ (n = 9)	TNBC (n = 23)	
Age at diagnosis					
Median (range)	69 (45–87)	64 (29–86)	59 (53–85)	64 (35–80)	66 (25–87)
ER					
0%	0 (0.0%)	0 (0.0%)	8 (88.9%)	23 (100.0%)	31 (30.7%)
>0%	22 (100.0%)	47 (100.0%)	1 (11.1%)	0 (0.0%)	70 (69.3%)
PR					
0%	0 (0.0%)	6 (12.8%)	8 (88.9%)	23 (100.0%)	37 (36.6%)
>0%	22 (100.0%)	41 (87.2%)	1 (11.1%)	0 (0.0%)	64 (63.4%)
Histotype					
Ductal	21 (95.5%)	41 (87.2%)	9 (100.0%)	21 (91.4%)	92 (91.0%)
Ductal/lobular	1 (4.5%)	3 (6.4%)	0 (0.0%)	0 (0.0%)	4 (4.0%)
Lobular	0 (0.0%)	3 (6.4%)	0 (0.0%)	1 (4.3%)	4 (4.0%)
Other (medullary, metaplastic)	0 (0.0%)	0 (0.0%)	0 (0.0%)	1 (4.3%)	1 (1.0%)
Grading					
G1	7 (31.8%)	2 (4.3%)	0 (0.0%)	0 (0.0%)	9 (8.8%)
G2	13 (59.1%)	19 (40.4%)	0 (0.0%)	1 (4.3%)	33 (32.7%)
G2/3	0 (0.0%)	3 (6.4%)	0 (0.0%)	0 (0.0%)	3 (3.0%)
G3	2 (9.1%)	23 (48.9%)	9 (100.0%)	22 (95.7%)	56 (55.4%)
Tumor size					
<1cm	5 (23.8%)	7 (14.9%)	3 (37.5%)	5 (25.0%)	20 (20.8%)
1–1.99 cm	8 (38.1%)	21 (44.7%)	1 (12.5%)	6 (30.0%)	36 (37.5%)
2–2.99 cm	2 (9.5%)	14 (29.8%)	0 (0.0%)	3 (15.0%)	19 (19.8%)
>=3 cm	6 (28.6%)	5 (10.6%)	4 (50.0%)	6 (30.0%)	21 (21.9%)
Unknown	1	0	1	3	5

ER: estrogen receptor; PR: progesterone receptor; TNBC: triple-negative breast cancer.

**Table 2:** Descriptive statistics of PSMA, TILs, and Ki67 by tumor subtype.

Marker	Luminal A Median (iqr) (1)	Luminal B Median (iqr) (2)	HER2+ Median (iqr) (3)	Triple-Negative Median (iqr) (4)	*p*-Value from Kruskal–Wallis’s Test	Post Hoc Comparison (Dunn’s Test)					
						2 vs. 1	3 vs. 1	4 vs. 1	3 vs. 2	4 vs. 2	4 vs. 3
	**N = 22**	**N = 47**	**N = 9**	**N = 23**							
PSMA	1.15 (0.5–2.4)	4.0 (2.2–5.3)	6.5 (3.4–8.5)	6.5 (5.4–7.7)	<0.001	<0.001	<0.001	<0.001	0.026	<0.001	0.169
Ki67	10.0% (6.0–17.0%)	28.0% (23.0–32.0%)	31.0% (28.0–42.0%)	48.0% (40.0–68.0%)	0.001	<0.001	<0.001	<0.001	0.107	<0.001	0.068
TILs	3.5% (2–5%)	7.0% (2–20%)	10.0% (5–20%)	15.0% (5–20%)	0.032	0.018	0.009	0.005	0.138	0.182	0.336

PSMA: prostate-specific membrane antigen; TILs: tumor-infiltrating lymphocytes; iqr: interquartile range; KW: Kruskal–Wallis test.

**Table 3:** Correlation analysis among PSMA, Ki67, and TILs.

Marker	Spearman’s Rho Coefficient (*p*-Value)	Spearman’s Rho Coefficient (*p*-Value) for the Different Tumor Subtypes			
		Luminal A (n = 22)	Luminal B (n = 47)	HER2+ (n = 9)	TNBC (n = 23)
PSMA vs. Ki67	0.695 (<0.001)	0.291 (0.189)	0.302 (0.039)	0.700 (0.035)	−0.076 (0.728)
PSMA vs. TILs	0.286 (0.003)	−0.466 (0.028)	0.108 (0.468)	0.591 (0.093)	0.124 (0.571)
KI67 vs. TILs	0.341 (<0.001)	−0.255 (0.251)	0.303 (0.038)	0.118 (0.762)	0.088 (0.689)

## Data Availability

All data generated or analyzed during this study are included in the published article. The raw data supporting the findings of this study are available from the authors upon reasonable request. Interested researchers may contact the corresponding authors to obtain access, subject to compliance with applicable data protection regulations and ethical guidelines.

## Supplementary Materials

The following supporting information can be downloaded at: https://www.mdpi.com/article/10.3390/biomedicines14030628/s1, Figure S1: Overall survival analysis of BC patients in relation to PSMA expression from Human Protein Atlas database; Figure S2: Overall survival analysis of BC patients in relation to Ki67 expression from KmPlotter.

## Abbreviations

The following abbreviations are used in this manuscript:

BC	Breast Cancer
PSMA	Prostate-Specific Membrane Antigen
TN	Triple-Negative
TILs	Tumor-infiltrating lymphocytes
TNBC	Triple-Negative Breast Cancer
ER	Estrogen Receptor
PR	Progesterone Receptor
HER2	Human epidermal growth factor receptor 2
LNs	Lymph nodes
