# Supplementary figures and images for: Prostate-Specific Membrane Antigen (PSMA): A Potential Theranostic Biomarker in Breast Cancer
Source: Biomedicines. 2026 Mar 11;14(3):628. doi: 10.3390/biomedicines14030628 (PMC13024703; doi:10.3390/biomedicines14030628)

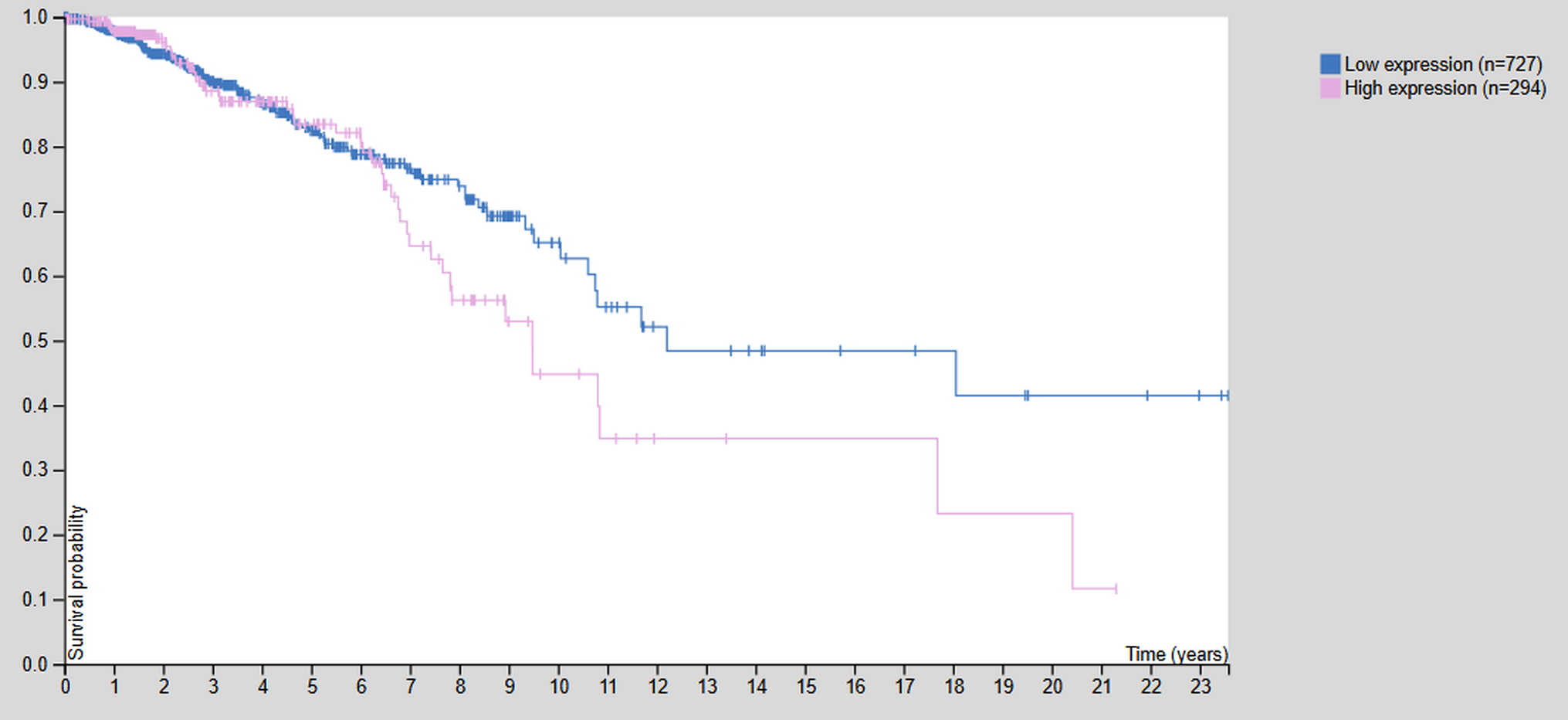

Supplement: Supplementary file 1 [file biomedicines-14-00628-s001.zip › Supplementary figure S1.PNG]

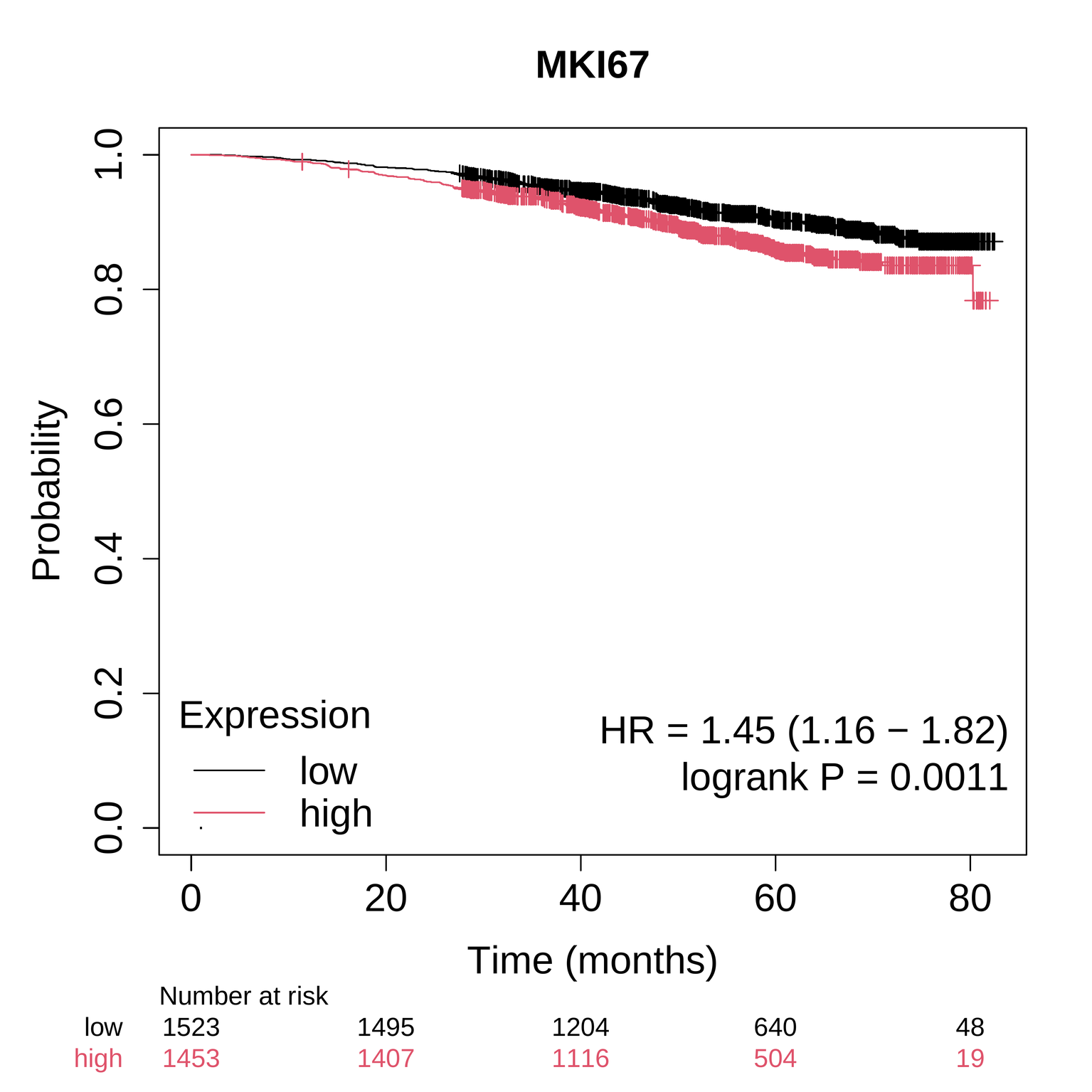

Supplement: Supplementary file 1 [file biomedicines-14-00628-s001.zip › Supplementary figure S2.png]
